# Innate and Adaptive Immunity Imbalance With Severe COVID-19 Pneumonia in Children and Adults

**DOI:** 10.3389/fped.2021.736013

**Published:** 2021-12-15

**Authors:** Zahra Shokati Eshkiki, Arman Shahriari, Maryam Seyedtabib, Mehdi Torabizadeh, Mohammad Ali Assarehzadegan, Roohangize Nashibi, Maryam Khosravi, Niloofar Neisi, Seyed Ali Mard, Ali Akbar Shayesteh

**Affiliations:** ^1^Alimentary Tract Research Center, Clinical Sciences Research Institute, Ahvaz Jundishapur University of Medical Sciences, Ahvaz, Iran; ^2^Department of Biostatistics and Epidemiology, School of Public Health, Ahvaz Jundishapur University of Medical Sciences, Ahvaz, Iran; ^3^Golestan Hospital, Clinical Research Development Unit, Ahvaz Jundishapur University of Medical Sciences, Ahvaz, Iran; ^4^Immunology Research Center, Institute of Immunology and Infectious Diseases, Iran University of Medical Sciences, Tehran, Iran; ^5^Infectious and Tropical Diseases Research Center, Health Research Institute, Ahvaz Jundishapur University of Medical Sciences, Ahvaz, Iran; ^6^Department of Immunology, School of Medicine, Ahvaz Jundishapur University of Medical Sciences, Ahvaz, Iran

**Keywords:** SARS-CoV-2, COVID-19, immune response alteration, innate immune cells, adaptive immune cells

## Abstract

**Introduction:** Little is known about the laboratory and radiological characteristics and clinical significance of peripheral immune alterations in patients with coronavirus disease 2019 (COVID-19). This study aims to clarify these aspects in children and adults with COVID-19.

**Methods:** In this consecutive pilot study, COVID-19 patients with the confirmed pneumonia and real-time RT-PCR were recruited prospectively in June 2020. The clinical, chest CT, and laboratory features, such as lymphocyte subpopulations, were analyzed for each individual.

**Results:** Forty confirmed COVID-19 patients, 11 severe children, 12 severe adults, and 17 critical adult patients, besides 20 healthy pediatrics and 14 healthy adults as controls, were enrolled prospectively. Adult patients, especially critical ones, had a much higher prevalence of laboratory and chest CT abnormalities. Data regarding immune cell subsets in children patients, compared with matched controls, had higher CD3+ CD8+ T cells (*p* = 0.004) and lower CD4+/CD8+ ratio (*p* = 0.042), while adult patients, compared with matched controls, had lower CD14+ monocytes (*p* = 0.032). Adult patients were also categorized as experiencing critical or severe illness on admission and, compared with severe patients, had lower total lymphocytes (*p* < 0.047), CD3+ T-lymphocytes (*p* < 0.002), and CD3+ CD8+ T cells (*p* = 0.001) and, on the other hand, had higher CD3+ CD4+ T cells (*p* = 0.012) and CD4+/CD8+ ratio (*p* = 0.003). Non survived adults, compared with survived patients, had significantly lower CD3+ T-lymphocyte (*p* = 0.005).

**Conclusion:** Unlike adult patients, who compared with matched controls and had more comorbidities, higher frequency of severe clinical symptoms, laboratory abnormalities, and immune cells alteration, clinical manifestations of COVID-19 in children (compared with matched controls) were relatively mild, and fewer clinical complications were seen either, perhaps because of a milder inflammatory response following their peripheral innate and adaptive immune cell alteration pattern.

## Introduction

Severe acute respiratory syndrome coronavirus 2 (SARS-CoV-2), a new strain of betacoronavirus, affects about 165 million people worldwide and over 2,700,000 Iranians from early 2020 (1). Curiously, it is reported that 18–30% of the affected people may be asymptomatic, while others show mild to severe to critical symptoms of SARS-CoV-2 infection (2). There are several risk factors related to COVID-19 severity, but the crucial factors could be age, health condition (history of diabetes and cardiovascular and pulmonary disease), and immune system function that seems to affect the morbidity and mortality of COVID-19 (3).

All people, both children and adolescents, are susceptible to this virus, and due to human-human transmission of this highly infectious virus, the increasing rate of SARS-CoV-2 morbidity and mortality has been observed today (4). However, it seems children are less susceptible to COVID-19 and the possible mechanisms of the resistance are controversial (5), possibly due to the ability of their B cells, when compared with B cells from adults, in production of natural antibodies (IgA, IgM, and IgG) promptly on encounter with new pathogens (6) or the lower binding ability of their ACE2 receptor (7).

As we know now, one of the most leading causes of mortality in both children and adult is COVID-19 pneumonia (8) because distinct immunological responses to viral infections can exist in each of which and result in severe damage to vital organs, such as pulmonary, especially in adult patients (9). Aberrant immune responses include lymphopenia, particularly T lymphocytes (both CD4+ T and CD8+ T cells), cytokine storm, and hyper-inflammatory responses that play a critical role in the pathology of SARS-CoV-2 (1, 10). Several previous studies have indicated an apparent decrease in peripheral lymphocytes of COVID-19 patients, but apparent alterations in the subsets, in both adult and children patients with COVID-19, were still unknown (2, 11).

Nowadays, other variants of the SARS-CoV-2 coronavirus that are different from the version first detected in China were detected. It is proved that those new variants carry higher transmissibility, but there is not enough evidence to be more lethal than the original one. Those new strains may have the ability to re-infect people who have received coronavirus vaccines or recovered from earlier versions of the coronavirus (10). Therefore, to better manage patients with COVID-19, it is crucial to elucidate the exact role of innate and adaptive immune cells in COVID-19 immunity induction and also in the boost of COVID immunity in the face of new variants.

There are many studies concerning changes of innate and adaptive immune cells and their correlation with the severity and outcome of the COVID-19 in adult patients (12–14). Nevertheless, there are not enough studies about these changes in pediatric patients. Here, for proper prognosis and management of this illness, we aimed to identify the clinical and immunological characteristics of both children and adult patients, compared with their age-matched and gender-matched healthy controls.

## Methods

### Study Population

In this prospective pilot study, we enrolled 40 COVID-19 patients (11 severe children, 12 severe adults, and 17 critical adult patients at Ahvaz Abuzar Children University Hospital and Ahvaz Razi University Hospital), with the confirmed pneumonia and real-time RT-PCR, in June 2020. We screened all patients who were initially admitted to the mentioned hospitals and had pneumonia, fever, cough, and radiographic presentation at the initial assessment or who had close contact with an infected individual within 14 days. We also excluded all COVID-19 patients who were receiving steroid medication. The diagnosis of COVID-19 was based on using a SARS-CoV-2 nucleic acid detection kit according to the manufacturer's protocol for detecting SARS-CoV-2 RNA in the throat or upper nasopharyngeal swab samples. According to the clinical findings (severity of pneumonia, respiratory failure, shock, and other organ failures), the severity of the disease was categorized (15). Severe and critical types were also defined according to these criteria: (1) breathing rate ≥30 times/min; (2) oxygen saturation (SpO_2_) ≤93% at rest; and (3) ratio of the partial pressure of arterial oxygen (PaO_2_) to the fraction of inspired oxygen (FiO_2_) ≤ 300 mmHg (16).

This study received ethical approval (IR.AJUMS.REC.1399.081) from the Ahvaz-Jundishapur University of Medical Sciences in Ahvaz, Iran. Written informed consent was obtained from adult participants or parents of enrolled children, and the study was carried out following the guidelines of the Declaration of Helsinki.

### Procedures

#### Data Collection

Sample collection, RT-PCR (PCR-Florescence Probing kit, Sansure Biotech Inc, China), and interpretation of results were made as previously described by Wang et al. (17). The following information on each patient was collected for all patients: age, sex, severity assessment on admission, symptoms, medical history, underlying comorbidities, exposure history, chest computed tomography (CT) or radiograph findings, laboratory findings, and therapeutic principles [comprising antiviral therapy (according to a defined clinical–pharmacologic protocol), monitoring of lung, kidney, liver, and myocardial functions; oxygen uptake if necessary; and active control over high fever]. On admission, patients were also assessed for their clinical type, according to guidelines for scoring adolescent and pediatric patients with COVID-19. We followed subjects for 2 weeks, and finally, patients' outcomes were considered as cured or failed (referred to death).

#### Flow Cytometry

Lymphocyte subset percentages were analyzed before initial treatment. EDTA anticoagulated peripheral blood (2 ml) were collected from patients with COVID-19 on admission (18). All samples were tested within 6 h of being obtained. Briefly, CD3+ total T-lymphocytes, CD19+ B-cell, CD3+/CD4+/CD8+ T-cell, and CD16+CD56+ NK-cell counts (cells/μl) were measured by multiple-color flow cytometry with human monoclonal anti-CD19-PE, anti-CD3-fluorescein isothiocyanate (FITC), anti-CD4-phycoerythrin (PE), anti-CD8-allophycocyanin (APC), anti-CD16-APC, and anti-CD56-PE antibodies (BD Multitest) according to the manufacturer's instructions. The cells were analyzed on a Partec Cube 6 Flow cytometry system (Sysmex Europe GmbH, Germany).

### Statistical Analysis

For analysis of adult data, once, we analyzed severe adults data and critical adults data separately, and then we pooled both of those data, as adults, and analyzed it again. We defined continuous data as mean, median, and SD, and categorical data were shown as numbers (%). Means of continuous variables between two groups were analyzed with parametric and non-parametric analysis methods such as independent group *t*-tests and Mann–Whitney *U*-test. All statistical analyses were done with SPSS Statistics version 16. Two-sided *p* < 0.05 were considered statistically significant.

## Results

### Demographic, Clinical, and Paraclinical Characteristics of Patients With COVID-19

The pilot study population consisted of 40 hospitalized COVID-19 patients with confirmed infection. During June 2020, 11 children (aged 1–16 years; all of them were severe cases, median 6 years) and 29 adults (aged ≥ 17 years; median in 12 severe cases 53 years and in 17 critical cases 70 years) COVID-19 cases were recruited prospectively in this pilot study. The severe/critical form of the COVID-19 was more common in males than females (54.5% of severe children, 75% of severe adults, and 47.1% of critical adult patients were male). Twenty healthy pediatrics and 14 healthy adults as age-matched and gender-matched healthy controls were included too.

Generally, seven types of abnormal radiographic presentations were seen: ground-glass opacity, linear opacity, patchy opacity, atelectasis, pleural effusion, mild segmental atelectasis, and consolidation. The most common radiographic findings of children, as severe cases, was linear opacity (27.27%). There was not any comorbidity in children patients. The most common symptoms in children were fever (72.73%), chills (72.73%), and dry cough (54.55%). The most common radiographic findings of adult patients were ground-glass opacity (severe cases 100%, critical cases 100%). The common comorbidity in adults was hypertension (severe cases 41%, critical cases 41.1%) and diabetes (severe cases 25%, critical cases 41.1%). The most common symptoms in adults were dyspnea (severe cases 91.6%, critical cases 70.5%), fever (severe cases 50%, critical cases 47%), chills (severe cases 50%, critical cases 47%), anorexia, and lethargy (in severe cases 50%) (Table 1).

**Table 1:** Clinical characteristics of COVID-19 patients.

**Characteristic**	**S.Ch**	**S.Ad**	**C.Ad**
**Symptoms**			
Fever	8 (72.73%)	6 (50%)	8 (47%)
Chills	8 (72.73%)	6 (50%)	8 (47%)
Weakness	4 (36.36%)	2 (16.6%)	5 (29.4%)
Lethargy	4 (36.36%)	6 (50%)	5 (29.4%)
Myalgia	–	5 (41%)	–
Dyspnea	1 (9.09%)	11(91.6%)	12 (70.5%)
Tachypnea	1 (9.09%)	–	–
Sore throat	1 (9.09%)	–	–
Dry cough	6 (54.55%)	5 (41%)	5 (29.4%)
Anorexia	1 (9.09%)	6 (50%)	5 (29.4%)
Abdominal pain	1 (9.09%)	1 (8.3%)	–
Nausea and vomiting	2 (18.18%)	3 (25%)	2 (11.7%)
Diarrhea	–	1 (8.3%)	–
Ischemia	1 (9.09%)	–	1 (5.8%)
Headache	–	–	2 (11.7%)
**Radiography**			
Ground-glass opacity	1 (9.09%)	12 (100%)	17 (100%)
Linear opacity	3 (27.27%)	–	–
Patchy opacity	1 (9.09%)	9 (75%)	12 (70.5%)
Atelectasis	1 (9.09%)	–	–
Pleural effusion	1 (9.09%)	–	–
Mild segmental atelectasis	1 (9.09%)	–	–
Consolidation	–	1 (8.3%)	9 (52.9%)
**Comorbidities**			
Malignancy	–	–	2 (11.7%)
Hypertension	–	5 (41%)	7 (41.1%)
Diabetes	–	3 (25%)	7 (41.1%)
Cardiovascular disease	–	1 (8.3%)	3 (17.6%)
Chronic kidney disease	–	–	1 (5.8%)
Pulmonary disease	–	1 (8.3%)	1 (5.8%)
Cerebrovascular disease	–	–	1 (5.8%)
Mortality rate	1 (9.09%)	1 (8.3%)	11 (64.7%)

*COVID-19,coronavirus 2019; S.Ch, severe child; S.Ad, severe adult; C.Ad, critical adult*.

*Data are the frequency of individuals (percentage)*.

Laboratory findings were completely different between severe and critical adult groups as the most important laboratory test findings of critical adults, compared with severe adults, were elevation of PT (*p* = 0.011), INR (*p* = 0.011), LDH (*p* = 0.016), CPK (*p* = 0.013), D-Dimer (*p* = 0.010), CRP (0.046), BUN (*p* = 0.000), WBC (*p* = 0.000), NLR (*p* = 0.42), RDW-CV (*p* = 0.009), and depletion of lymphocytes (*p* = 0.04) (Table 2).

**Table 2:** Comparison of peripheral variables of the severe adult with critical adult patients.

**Variables**	**S.Ad** **= 12*****n***		**C.Ad = 17** ***n***		***P*** **-value**
	**Male =** ***9n*** **(75%)**	**Female = 3*****n*** **(25%)**	**Male = 8*****n*** **(47.1%)**	**Female = 9*****n*** **(52.9%)**	
	**Median (IQR)**	**Mean ± SD**	**Median (IQR)**	**Mean ± SD**	
Age, years		59.33 ± 17.35		63.76 ± 14.55	Φ 0.462
**Laboratory findings**				
WBC (10^9^/L)	5.85 (4.3)		10.40 (12)		Ω 0.000^***^
Lym (10^9^/L)	21.00 (11.5)		18.00 (23)		Ω 0.047^*^
NLR	13.20 (6)		14.30 (13.20)		Ω 0.042^*^
CRP (mg/L)	31.50 (50)		87.50 (46)		Ω 0.046^*^
D-Dimer (μg/ml)	1.10 (1.03)		2.90 (3.70)		Ω 0.010^*^
Neu (10^9^/L)		72.50 ± 7.66		89.13 ± 9.75	Φ 0.063
CD4+/CD8+ ratio	1.04 (0.43)		2.00 (1.15)		Ω 0.003^**^
CD19+ B cell (%)	9.50 (6)		11.00 (25)		Ω 0.128
CD14+ monocyte (%)	5.50 (2.3)		5.00 (7.0)		Ω 0.146
CD3+ Total T lymphocyte (%)		70.17 ± 9.36		55.69 ± 11.85	Φ 0.002^**^
CD3+CD4+ T cell (%)		50.83 ± 10.88		62.00 ± 11.05	Φ 0.012^*^
CD3+CD8+ T cell (%)		42.25 ± 9.56		28.59 ± 9.17	Φ 0.001^**^
CD16+CD56+ Nk cell (%)		6.67 ± 5.18		10.82 ± 8.81	Φ 0.155

*Data are presented as median (IQR) and mean ± SD*.

*COVID-19, coronavirus 2019; S.Ad, severe adult; C.Ad, critical adult; WBC, white blood cell; Neu, neutrophil; Lym, lymphocyte; NLR, neutrophil to lymphocyte ratio; CRP, c-reactive protein; D-Dimer, a fibrin degradation product or FDP. Ω, Mann–Whitney U; Φ, T-test*.

* *p < 0.05*;

** *p < 0.01*;

*** *p < 0.001*.

### Peripheral Innate and Adaptive Immune Cells of COVID-19 Patients

We initially analyzed the levels of innate and adaptive immune cell subsets by flow cytometry in whole blood. The mean time of onset of symptoms to testing of immune cell subsets was 8.8 ± 1.2 days.

**Figure 1:**
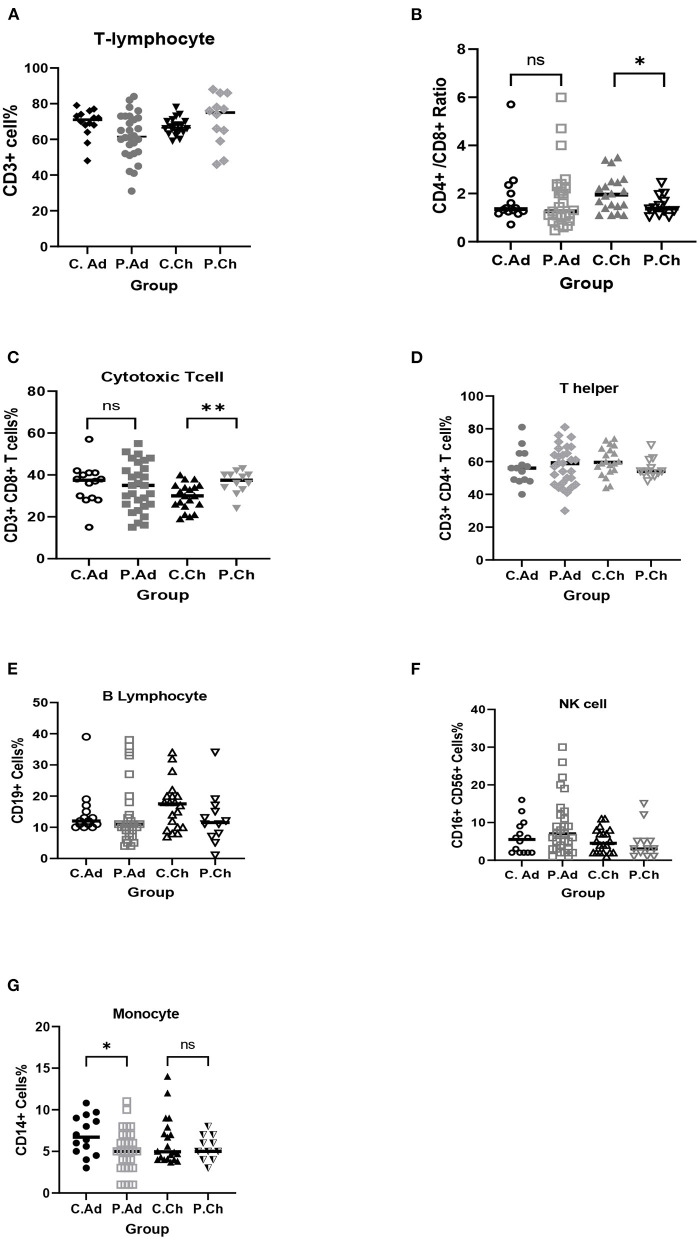
Comparison of peripheral innate and adaptive immune cells of COVID-19 children patients (P.Ch) with matched-healthy control (C.Ch), and COVID-19 adult patients (P.Ad) with matched-healthy control (C.Ad). **(A)** Total T-lymphocytes (CD3+), **(B)** CD4+/CD8+ ratio, **(C)** Cytotoxic T cells (CD3+, CD8+), **(D)** T helper (CD3+, CD4+), **(E)** B lymphocytes (CD19+), **(F)** NK cells (C16+, CD56+), **(G)** Monocyte (CD14+). ^*^p < 0.05; ^**^p < 0.01; NS, not significant.

Children with COVID-19 had significantly higher CD3+ CD8+ T cells (*p* = 0.004) and, on the other hand, had significantly lower CD4+/CD8+ ratio (*p* = 0.042) compared with age-matched and gender-matched healthy children (Table 3; Figure 1).

**Table 3:** Comparison of peripheral innate and adaptive immune cells of COVID-19 children patients with healthy children.

**Variables**	**Child (control) = 20** ***n***		**Child (case) = 11** ***n***		***P*** **-value**
	**Male = 13*****n*** **(65%)**	**Female = 7*****n*** **(35%)**	**Male = 6*****n*** **(54.5%)**	**Female = 5*****n*** **(45.5%)**	
	**Median (IQR)**	**Mean ± SD**	**Median (IQR)**	**Mean ± SD**	
Age, years		3.70 ± 2.93		6.19 ± 5.00	Ω 0.11
CD3+ total T lymphocyte (%)		67.10 ± 4.71		70.75 ± 14.19	Φ 0.295
CD3+CD8+ T cell (%)		29.60 ± 6.38		36.50 ± 5.40	Φ 0.004^**^
CD4+/CD8+ ratio		2.02 ± 0.77		1.50 ± 0.44	Φ 0.042^*^
CD3+CD4+ T cell (%)	59.50 (14)		54.00 (8)		Ω 0.091
CD16+CD56+ Nk cell (%)	4.50 (6)		3.00 (4)		Ω 0.431
CD19+ B cell (%)	17.50 (10)		11.50 (9)		Ω 0.157
CD14+ monocyte (%)	4.95 (3.7)		5.00 (3)		Ω 0.855

*Data are presented as median (IQR) and mean ± SD*.

Ω, *Mann–Whitney U; Φ, T-test*.

* *p < 0.05*;

** *p < 0.01*.

Adult COVID-19 patients had significantly lower CD14+ monocytes (*p* = 0.032) than age-matched and gender-matched healthy adults (Table 4; Figure 1).

**Table 4:** Comparison of peripheral innate and adaptive immune cells of COVID-19 adult patients with healthy adults.

**Variables**	**Adult (control) = 14**		**Adult (case) = 29**		***p*** **-value**
	**Male = 7*****n*** **(50%)**	**Female = 7*****n*** **(50%)**	**Male = 17*****n*** **(58.60%)**	**Female = 12*****n*** **(41.40%)**	
	**Median (IQR)**	**Mean ± SD**	**Median (IQR)**	**Mean ± SD**	
Age, years		51.50 ± 9.53		58.93 ± 13.29	Φ 0.069
CD3+CD4+ T cell (%)		56.93 ± 10.71		57.38 ± 12.15	Φ 0.906
CD3+CD8+ T cell (%)		35.71 ± 9.63		34.24 ± 11.44	Φ 0.680
CD14+ monocyte (%)		6.92 ± 2.38		5.10 ± 2.58	Φ 0.032*
CD3+ Total T lymphocyte (%)	71.00 (8)		61.50 (21)		Ω 0.065
CD4+/CD8+ ratio	1.36 (0.86)		1.26 (1.15)		Ω 0.371
CD16+CD56+ Nk cell (%)	5.50 (7)		7.00 (10)		Ω 0.268
CD19+ B cell (%)	12.00 (5)		11.00 (10)		Ω 0.155

*Data are presented as median (IQR) and mean ± SD*.

Ω, Mann–Whitney U; Φ, T-test. ^**^p < 0.01.

**Figure 2:**
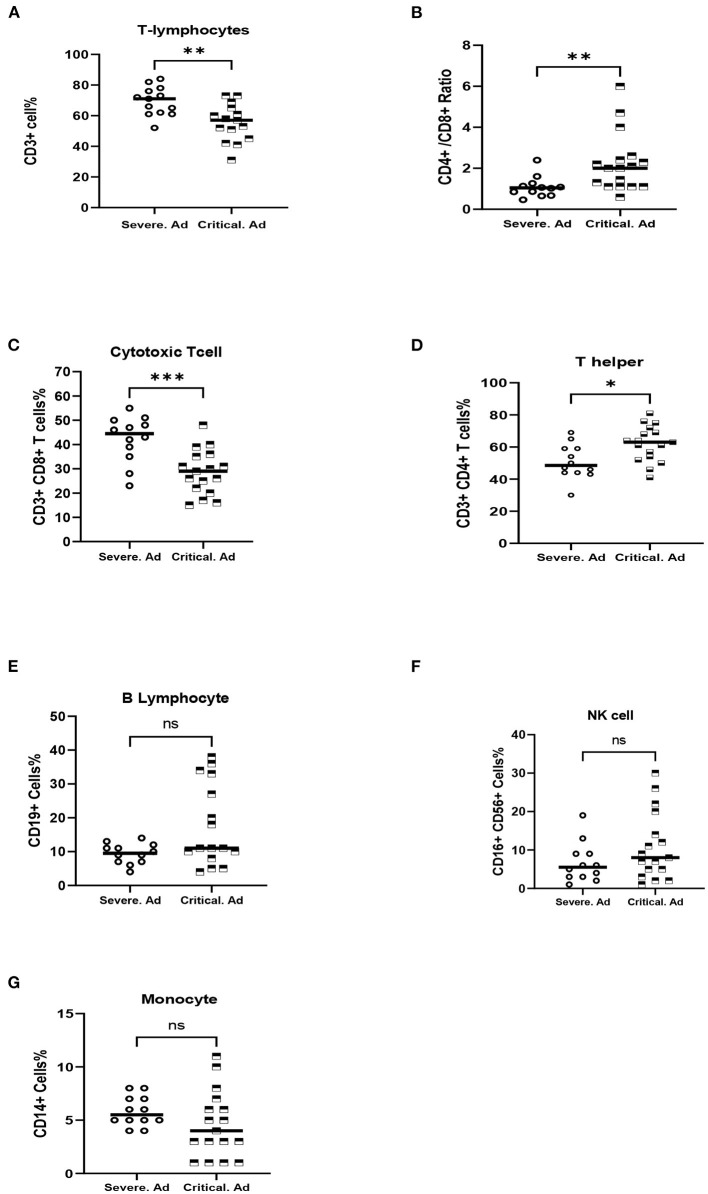
Comparison of peripheral innate and adaptive immune cells of severe COVID-19 adult patients (Severe.Ad) with critical COVID-19 adult patients (Critical.Ad). **(A)** Total T-lymphocytes (CD3+). **(B)** CD4+/CD8+ ratio, **(C)** Cytotoxic T cells (CD3+, CD8+). **(D)** T helper (CD3+, CD4+). **(E)** B lymphocytes (CD19+). **(F)** NK cells (C16+, CD56+). **(G)** Monocyte (CD14+). ^*^p < 0.05; ^**^p < 0.01; ^***^p < 0.001; NS, not significant.

As described before, adult patients were categorized as experiencing critical or severe illness on admission. Critical cases had significantly lower CD3+ T-lymphocyte (*p* = 0.002) and CD3+ CD8+ T cells (*p* = 0.001) and also had significantly higher CD3+ CD4+ T cells (*p* = 0.012) and CD4+/CD8+ ratio (*p* = 0.003) compared to patients with severe illness (Table 2; Figure 2).

Interestingly, non-survived adults had significantly lower CD3+ T-lymphocyte (*p* = 0.005) than survived adult patients (Table 5).

**Table 5:** Comparison of peripheral innate and adaptive immune cells of COVID-19 survived patients with non-survived cases.

**Variables**	**Survived = 17** ***n***		**Non-survived = 12** ***n***		***P*** **-value**
	**Severe = 11*****n*** **(64.7%)**	**Critical = 6*****n*** **(35.30%)**	**Severe = 1*****n*** **(8.3%)**	**Critical = 11*****n*** **(91.7%)**	
	**Median (IQR)**	**Mean ± SD**	**Median (IQR)**	**Mean ± SD**	
Age, years		60.80 ± 16.42		63.39 ± 14.78	Φ 0.546
CD3+ Total T Lymphocyte (%)		67.18 ± 11.20		53.73 ± 11.36	Φ 0.005^**^
CD3+CD4+ T cell (%)		54.53 ± 12.49		61.42 ± 10.88	Φ 0.135
CD3+CD8+ T cell (%)		37.35 ± 11.94		29.83 ± 9.48	Φ 0.081
CD14+ Monocyte (%)		5.82 ± 2.22		4.08 ± 2.81	Φ 0.073
CD16+CD56+ NK cell (%)		7.29 ± 6.17		11.67 ± 9.11	Φ 0.134
CD4+/CD8+ Ratio	1.10 (0.58)		2.05 (1.31)		Ω 0.080
CD19+ B cell (%)	10.0 (3)		11.00 (25)		Ω 0.329

*Data are presented as median (IQR) and mean ± SD*.

*Ω, Mann–Whitney U; Φ, T-test*.

** *p < 0.01*.

## Discussion

COVID-19 is an emerging contagious disease with a high prevalence of pneumonia in infected patients. For precise diagnosis and effective treatment of this highly infectious disease, we need to understand the differences between immunological and clinical features in both adult and children patients. Importantly, our study showed that the immunological and clinical feature changes in adults and children COVID-19 patients were quite different. Moreover, the main clinical, immunological, and paraclinical feature changes of COVID-19 in adults with critical illness were utterly different from adults with severe illness.

As we expected (9), adult patients with critical illnesses, compared to severe ones, had more common comorbidities such as hypertension, diabetes, or cardiovascular disease. Furthermore, adults in the critical group had a higher prevalence of symptoms such as dyspnea. While children did not have any comorbidity, in agreement with a previous study, fever, chills, and dry cough were the most common symptoms in children with COVID-19 infection (19). A recent case-control study, which evaluated risk factors of death in 172 COVID-19 patients, included 32 nonsurvived cases and has reported age, comorbidities, dyspnea, and chest pain at admission as the main factors associated with a higher risk of death in COVID-19 patients (20). The most CT abnormality in children was also linear opacity, and the prevalence of abnormalities on chest CT such as ground-glass opacity and patchy opacity was much higher in adult COVID-19 patients, mainly from the critical group. In line with a recent study (21), our data showed that children do not share the same imaging pattern as adults that is also an important issue to show the severity of COVID-19.

The immune system (including innate immune cells, monocytes, macrophages, and granulocytes, and also adaptive immune cells, including T and B lymphocytes) plays a critical role in combating COVID-19. There are many underway efforts toward understanding immune cell responses, reactions, and alterations during COVID-19 infection (22).

Several recent genetic discoveries revealed the crucial role of innate immunity in determining COVID-19 severity and outcome. In a recent case series, Van Der Made et al. investigated the primary genetic and immunological condition of four severe young patients with COVID-19 from two families to characterize any immune defects. They reported rare loss-of-function variants of the X-chromosomal Toll-like receptor 7 (TLR7), with immunological defects in type I and II interferon production probably due to the aberrant function of peripheral blood mononuclear cells in severe COVID-19 patients (23). Zhang et al. also have identified enrichment in rare loss-of-function variants underlying autosomal-dominant or autosomal-recessive deficiencies at the 13 human loci, in 23 patients of 659 patients with life-threatening COVID-19 pneumonia. They revealed how inborn errors at the 13 human loci, which are known to govern TLR3- and IRF7-dependent type I IFN immunity, can lead to life-threatening pneumonia in COVID-19 patients (24). Recently, Schultze et al. (22) proved that dysregulation in mononuclear phagocyte response to SARS-CoV-2 is associated with severe COVID-19. In line with those findings, our results demonstrated that in adult patients, compared with matched controls, CD14+ monocytes significantly decreased, while there was not any significant difference in children patients compared with matched controls. This finding can indicate more severity of COVID-19 infection in adult patients.

Our results showed that lymphopenia is common in all adult patients with COVID-19, especially in critical adults, indicating an impairment of the immune system following SARS-CoV-2 infection. Surprisingly, the prevalence of lymphopenia in critical adults was not only significantly higher than severe cases, but also NLR in this group was significantly higher than severe ones. Recently, Yang et al. (25) reported lymphopenia in 80% of critically ill adults, whereas Chen et al. (11) reported lymphopenia only in 25% of mild COVID-19 cases, suggesting that lymphopenia is correlated with the severity of COVID-19. Lymphopenia might be occurred by inflammatory mediators, virus attachment, or transudation of circulating lymphocytes into inflammatory lung tissues (26). Therefore, lymphopenia is a severity indicator for COVID-19.

Lymphocyte subsets, compared with innate immune cells, play an essential role in maintaining immune system defense in infectious diseases, like viral infections, which can dysregulate the levels of lymphocyte subsets (26, 27). Lymphocyte subsets are involved in cellular and humoral immunity. Therefore, it is essential to elucidate the lymphocyte subsets alteration in COVID-19 and clarifying the immune mechanisms against SARS-CoV-2. Importantly, our results demonstrated that not only nonsurvived adults and critical adult patients had a significant decrease in CD3+ Total T-lymphocytes (compared with survived patients and severe adults, respectively) but also in critical adults, CD3+ CD8+ T cells were decreased significantly. On the other hand, our data clarified that children had a significant increase in CD3+ CD8+ T cells and also significantly decreased CD4+/CD8+ ratio than matched controls. Recent studies suggested that decreased CD3+ CD8+ T cells are a potential predictor of COVID-19 severity (26). Moreover, recent researches have proved the role of CD3+ CD8+ T cells in mediating viral clearance of respiratory infections such as influenza A virus (IAV), human metapneumovirus, and respiratory syncytial virus (RSV) (28, 29). Interestingly, in animal studies, the transfer of IAV-immune or RSV– CD8+ T cells significantly reduced viral titers in athymic mice (30, 31).

As we expected, among adult patients, critical cases significantly had a higher level of CD3+ CD4+ Th cells and CD4+/CD8+ ratio than severe ones. As we know, the level of proinflammatory cytokines, such as IL-6, in critically ill patients increases sharply, which is called “cytokine storm.” It has been shown that IL-6, during germinal center formation, may increase inflammation in COVID-19 patients. Therefore, IL6, as a proinflammatory cytokine, is one of the main factors of cytokine storm syndrome, and the serum levels of IL-6 in COVID-19 patients are positively correlated with the severity of COVID-19 (32). This hypercytokinemia not only indicates a poor prognosis in COVID-19 but also results in infiltration of CD3+ CD4+ Th and macrophages as proinflammatory cells and contributes to the higher mortality of critical patients with COVID-19 (33). Thus, an increase in CD3+ CD4+ Th cells is positively correlated with the severity of COVID-19.

We also observed that critical adults compared with severe adult patients had a much higher prevalence of laboratory abnormalities such as increased WBC, NLR, CRP, and D-dimer and conversely decreased total lymphocytes (*p* < 0.05). Recent studies have proved that COVID-19 patients with more severe illness tended to have higher NLR, which has served as a marker of subclinical inflammation and could use as a predictor of in-hospital death in COVID-19 patients (34). This marker was significantly higher in critical adults than severe adult patients. CRP and ESR are known as markers of inflammation. Lastly, Akbari et al. (35) found that CRP and ESR could serve as reliable severity indicators of COVID-19. D-dimer as a coagulation indicator could also reflect the severity of the infection. COVID-19 might be complicated with coagulopathy, and it shows that this coagulation dysfunction correlates with adverse clinical outcomes in COVID-19 patients. Elevation in D-dimer level, as a fibrin degradation product (FDP), is the most common pattern of coagulopathy in COVID-19 (36). Based on the results of a comprehensive study (37), there are fewer laboratory abnormalities in severe adults than critical ones, suggesting more minor immune damage and a much milder immunological response in those COVID-19 patients.

Taking all these findings into consideration, it seems COVID-19 in children (38) and also, as expected, in severe adult patients than critical cases is pretty mild in terms of presenting symptoms.

### Limitations

The major limitation was the small sample size. We conducted this study at the beginning of the COVID-19 pandemic, which less commonly involved the pediatric age group. We did not have either critical pediatric patients during the study period. The parents of children patients were not also interested that their kids were taking part in the study. We had a lack of healthy adults and children who were willing to participate in our study in this novel pandemic. Another limitation was the difference in age between children (1–16 years, median 6 years), which may interfere with the immune response of different ages. We also had no prior information of cases (clinical history and laboratory findings).

## Conclusion

In conclusion, our study showed that peripheral lymphocyte subset alteration showed a clear association with the clinical characteristics of COVID-19. In pediatric patients, compared with matched controls, fever, chills, and dry cough are common manifestations. Most of the children have neither abnormal radiological findings nor obvious symptoms and comorbidity history or any laboratory abnormality, which can indicate that children could be relatively mild cases, rather than adults. On the other hand, adult patients, compared with matched controls, had more common comorbidities and higher prevalence of clinical symptoms, laboratory abnormalities, and immune cell alteration, which may have resulted in more severe cases. As explained, CD3+ CD8+ T also tended to be an independent predictor for COVID-19 severity and therapeutic targets differently. Therefore, these findings might help elucidate the pathogenesis of SARS-CoV-2 and develop novel biomarkers and therapeutic strategies for COVID-19.

## Data Availability Statement

The original contributions presented in the study are included in the article/supplementary material, further inquiries can be directed to the corresponding author/s.

## Ethics Statement

The studies involving human participants were reviewed and approved by Ahvaz-Jundishapur University of Medical Sciences Ethics Committee Number: IR.AJUMS.REC.1399.081. Written informed consent to participate in this study was provided by the participants' legal guardian/next of kin.

## Author Contributions

ZS: conceived and designed the study. AS, MT, and MA: participated in sample collection and clinical follow-up of the patients. RN and MK: performed the experiments. MS, NN, and SM: analyzed and interpreted the data. AAS: managed the study. All authors approved the final version.

## Funding

This work was financially supported by Ahvaz Jundishapur University of Medical Sciences (RDC-9902).

## Conflict of Interest

The authors declare that the research was conducted in the absence of any commercial or financial relationships that could be construed as a potential conflict of interest.

## Publisher's Note

All claims expressed in this article are solely those of the authors and do not necessarily represent those of their affiliated organizations, or those of the publisher, the editors and the reviewers. Any product that may be evaluated in this article, or claim that may be made by its manufacturer, is not guaranteed or endorsed by the publisher.
